# Supplementary material for: Factors associated with non-adherence to Antiretroviral Therapy (ART) in adult individuals living with Human Immunodeficiency Virus (HIV) in Mogadishu, Somalia: A hospital-based cross-sectional study
Source: PLoS One. 2025 Apr 24;20(4):e0320983. doi: 10.1371/journal.pone.0320983 (PMC12021151; doi:10.1371/journal.pone.0320983)
Supplement: Attachment — Submitted filename: Reviewer_comments_declet_1.docx [file pone.0320983.s003.docx]

Thank you for giving me the chance to review this paper. I found the manuscript was dealt with one of the public health importance. This might help health policymakers and healthcare providers with an important clue.

Some of the concerns are included below

1. General

Paper needs language and grammatical edition

The manuscript has no line number to mention the issue easily

2. Abstract

Why unnecessary abbreviations in the Abstract? Rewrite them in full statement since it is conclusion it reduces its attractiveness to reader, may be only AOR.

The keywords: your keywords were about nine, better if you minimize it to the standard and add full forms of shortened words or acronyms and abbreviations.

3. Methods

Better if you add the health profession profile data and level of ART clinic services from the study area in related to your study problem

4. Inclusion/exclusion criteria

The author excluded incomplete charts, what are these incomplete charts? Better if operationalized such words

5. Sample size determination

The author included 453 participants after calculating sample size which was 398. What is your evidence to use 453 as a sample size? If so what is the importance to calculate sample size by using the proportion of the problem? Let the author describe his reasons

6. Data collection procedure

The author used both primary and secondary data, how could you manage the recall bias since you asked the previous history of remembered to take pills? Or you should include in the limitation of the study

7. Data Analysis procedure

The author said the multivariate analysis was carried out in order to identify factors associated with ART non-adherence. Do you think there could be confounding factors? If so how did you manage them? Did you check the multicholinerity or VIF? I need clarification

8. Results

From the beginning what is the significance of this study? Let me hear your new finding

Look at your descriptive part it should be modified to as of more attractive for readers and better if you describe 2/3 of your variables included in the defined table.

9. Discussion

Your justification needs strong modifications

It was not clear your justification was for which part

Add the general finding of these references you tried to compare your result to be clear for the readers, now it is not clear whether it is really higher, lower or comparable with yours.

Discuss for comparable results, higher results and lower results in different and your justification should be also for each difference. Even I couldn’t find another studies justification or support for your some variables eg, perceived stigma. Rewrite them

Generally the discussion part needs strong justifications

10. Conclusion:

The author concluded his findings by high burden of non-adhering to ART but didn’t describe his baseline to say high, I think as the author report there was no study result in the country with the same problem. Please add your baseline why say high burden on non-adherence to ART

11. References

Some of your references are outdated (13, 24, 25, 26 ....) please revise them

As a major

The issue of your sample size

You focused only single institution

Didn’t measure the confounding factors

The way you write discussion
