# Supplementary material for: Factors associated with non-adherence to Antiretroviral Therapy (ART) in adult individuals living with Human Immunodeficiency Virus (HIV) in Mogadishu, Somalia: A hospital-based cross-sectional study
Source: PLoS One. 2025 Apr 24;20(4):e0320983. doi: 10.1371/journal.pone.0320983 (PMC12021151; doi:10.1371/journal.pone.0320983)
Supplement: Attachment — Submitted filename: Point_by_point_response_auresp_2.docx [file pone.0320983.s005.docx]

| **Reviewer comment** | **Author Response** |
| --- | --- |
| **Dear reviewer,**  **Thank you for reviewing our manuscript and providing constructive comments. We greatly appreciate your valuable input.** | |
| Revise the grammar more. | Dear Reviewer/Editor, Thank you. I have reviewed the document line by line and corrected any errors observed, as per your suggestions. |
| Minimize the background from the abstract | Thank you. I also minimized as you suggested. |
| Again, look at your discussion and conclusion | Thank you. I reviewed and corrected erorrs. |
| The paper needs a language and grammatical edition. The manuscript has no line number to mention the issue easily. | Thank you, we've proofread the paper, consulted language experts for verification, and integrated the line numbers as per your recommendation. |
| **Abstract**  Why unnecessary abbreviations in the Abstract? Rewrite them in full statements since its conclusion reduces its attractiveness to the reader, maybe only AOR. | We rewrite them in full statements as you suggested, except for AOR and HIV words which are quite common. |
| **Keywords**  Your keywords were about nine, better if you minimize it to the standard and add full forms of shortened words or acronyms and abbreviations. | About the keywords, we minimized them as you suggested. |
| **Inclusion/exclusion criteria**  The author excluded incomplete charts, what are these incomplete charts? Better if operationalized such words. | Baseline incomplete medical record files were CD4 (cell/mm^3^), HB (g/dl), HIV Viral load (copies/mL), history of co-morbid disease, and history of opportunistic infections. **We added the paper following your suggestion** |
| **Sample size determination**  The author included 453 participants after calculating sample size which was 398. What is your evidence to use 453 as a sample size? If so what is the importance to calculate sample size by using the proportion of the problem? Let the author describe his reasons | We appreciate the reviewer's attention regarding the sample size. The primary aim of determining the sample size is to ensure a representative subset of the population for accurate outcome inference. However, using a sample larger than the minimum calculated size often enhances representativeness and consequently yields more precise results, especially considering this study is the first of its kind in the study area (doi: 10.4103/0974-7788.59946).  In our scenario, we had the necessary resources to study a sample size of up to 453, exceeding the minimum calculated size by only 55 cases. To the best of our knowledge, this marginal increment does not significantly impact the study's validity; rather, it bolsters the reliability of our findings and ensures a robust representation of the investigated population. |
| **Data collection procedure**  The author used both primary and secondary data, how could you manage the recall bias since you asked the previous history of remembered to take pills? Or you should include in the limitation of the study. | Dear Reviewer, Thank you for your valuable insights. We acknowledge your concerns regarding the recall bias. The approach we used aligns with several studies within our work. To clarify our assessment did not involve directly asking the participants about their past history of pill intake. Instead, **we quantified the number of pills taken within the last month from the prescribed medication by counting the remaining pills in the box and the number of ART pills previously dispensed individually.** This method aligns with the cited relevant literature aimed to mitigate potential recall biases [16,17,21-23].  Moreover, we used a well-structured questionnaire that underwent validation and reliability assessments, including the calculation of Cronbach's Coefficient alpha value and the IOC method. |
| **Data Analysis procedure and Confounding factors.**  The author said the multivariate analysis was carried out in order to identify factors associated with ART non-adherence. Do you think there could be confounding factors? If so how did you manage them? Did you check the multicholinerity or VIF? I need clarification. | We checked for confounding factors and multicollinearity by using the Variance Inflation Factor (VIF) in our multiple regression analysis. VIF values in our study ranged from 1.04 to 1.35 for all variables. A VIF value exceeding 10 indicates the presence of multicollinearity, while below 10, indicates no significant multicollinearity among the analyzed variables. To strengthen our analysis, we recorded detailed information on this process but couldn't include it in the article due to space limitations. However, we included it now as you suggested. |
| **Results**  From the beginning what is the significance of this study? Let me hear your new finding.  Look at your descriptive part it should be modified to as of more attractive for readers and better if you describe 2/3 of your variables included in the defined table. | This study's significance is paramount as it addresses the pressing issue of Antiretroviral Therapy (ART) non-adherence among individuals living with HIV in Mogadishu, Somalia, which is located in a region burdened by high HIV prevalence and inadequate healthcare coverage. By identifying factors contributing to ART non-adherence this research offers crucial insights. It fills a substantial knowledge gap in Somalia's context, informing targeted interventions and policy changes to enhance treatment outcomes, reduce mortality rates, and improve the overall quality of life for those living with HIV. Moreover, its implications extend globally, guiding similar initiatives in regions facing comparable challenges, thereby contributing significantly to the broader efforts of managing and mitigating the impact of HIV/AIDS worldwide. To the best of our knowledge, this is the first similar study conducted in Somalia.  **We will revise and readjust the descriptive part and we only interpreted significant variables as you suggested.** |
| **Discussion**  Your justification needs strong modifications. It was not clear your justification was for which part. Add the general finding of these references you tried to compare your result to be clear for the readers, now it is not clear whether it is really higher, lower or comparable with yours.  Discuss for comparable results, higher results and lower results in different and your justification should be also for each difference.  Even I couldn’t find another studies justification or support for your some variables eg, perceived stigma. Rewrite them.  Generally, the discussion part needs strong justifications. | We are thankful for the feedback received and have diligently revised the discussion section to ensure a more comprehensive and clearer justification of our findings, as you suggested. |
| **Conclusion:**  The author concluded his findings by high burden of non-adhering to ART but didn’t describe his baseline to say high, I think as the author report there was no study result in the country with the same problem. Please add your baseline why say high burden on non-adherence to ART. | We've considered your concern regarding this issue. To better reflect the findings accurately, we've revised the term 'high burden' to 'sub-optimal adherence.' This adjustment aims to align more closely with the study's results, ensuring a clearer representation of the situation regarding ART adherence.  Thank you for your valuable input. |
| **References**  Some of your references are outdated (13, 24, 25, 26 ....) please revise them. | We've revised all the references as mentioned, except for No. 24, which pertains to the Indexes of item-objective congruence (IOC) questionnaire validity. Unfortunately, we haven't found a comparable updated reference for this yet. |
| You focused only single institution. | The hospital involved in this study holds significance as a key teaching and referral institution, being the largest healthcare facility in the city and one of the two government-operated hospitals in Somalia. Notably, it boasts the region's most extensive ART (Antiretroviral Therapy) centre, catering to southern and central Somalia. Over 70% of the country's HIV-positive population receive care at this facility and have a documented medical history [16-18].  However, we acknowledge that including multiple centers in the study would have been advantageous, and we recognize this as one of the study's limitations. |

**Finally, I extend my heartfelt gratitude for taking the time to review our manuscript and for your insightful feedback. Your constructive comments have been immensely valuable to us, and we sincerely appreciate your thoughtful input. Thank you once again for your dedication and contributions to improving our work.**
